# Downregulated HIPK2 Expression in T Cells Is Associated with Occult Hepatitis B Virus Infection

**DOI:** 10.3390/microorganisms14071523

**Published:** 2026-07-12

**Authors:** Zhi-Hua Jiang, Mei-Lin Huang, Li-Ping Hu, Xue-Yan Wang, Qin-Yan Chen, Lu-Juan Zhang, Ning Zhang, Ying Huang, Tim J. Harrison, Ge Zhong, Zhong-Liao Fang

**Affiliations:** 1 Guangxi Key Laboratory for the Prevention and Control of Viral Hepatitis, Guangxi Zhuang Autonomous Region Center for Disease Prevention and Control, Nanning 530028, China; jiangzhihuagxcdc@163.com (Z.-H.J.);; 2 School of Preclinical Medicine, Guangxi Medical University, Nanning 530021, China; 3 Division of Medicine, University College London Medical School, London WC1E 6BT, UK

**Keywords:** hepatitis B virus, occult HBV infection, HIPK2 gene, downregulated expression

## Abstract

Occult hepatitis B virus (HBV) infection (OBI) has the same transmission routes and clinical outcomes as overt HBV infection. However, due to the lack of reliable biomarkers, its occurrence and potential clinical harm are often overlooked. We previously found that the prevalence of OBI increases with age among individuals vaccinated at birth, but the transcriptome characteristics of their immune cells remain unknown. In this study, fifteen subjects born between 1987 and 1993 and vaccinated perinatally were recruited. They were divided into OBI, inactive chronic HBsAg carriers (ICHC) and healthy control groups. Blood samples were drawn from each subject for scRNA-seq and subsequent validation studies. The results showed that eleven cell types and nine T-cell subsets were identified, with no significant differences in the proportions of T-cell subsets among the three groups. HIPK2 expression in T cells was downregulated 1.42-fold in the OBI group versus healthy controls (*p* = 2.01 × 10^−69^) and upregulated 1.34-fold versus the ICHC infection group (*p* = 9.95 × 10^−61^). Differentially expressed genes (DEGs) in immune-related pathways were enriched in the OBI group. TP53 plays a central role in HIPK2 function in the context of OBI. Serum HIPK2 levels were significantly lower in the OBI group than in the healthy controls (*p* = 0.047), but they were also significantly lower than in the ICHC infection group. In conclusion, downregulated HIPK2 expression in T cells is associated with OBI. The functional role of HIPK2 in OBI, including its role as a biomarker to distinguish OBI from a healthy status, warrants further investigation.

## 1. Introduction

Hepatitis B virus (HBV) infection can be classified as overt or occult, based on serological and molecular markers. The major difference is that in overt infection, HBsAg is positive, whereas in occult infection, HBsAg is negative but HBV DNA can be detected in serum and/or liver tissue [1].

Universal immunisation of newborns with hepatitis B vaccines has dramatically reduced the prevalence of overt HBV infection. Globally, the prevalence of persistent HBV infection among children below five years of age fell from 4.7% to 1.3% over two decades [2]. Immunisation has also reduced the incidence of hepatocellular carcinoma (HCC) in Taiwan [3] and elsewhere [4]. A remarkable reduction in HBV-related diseases was observed in Italy following the implementation of universal vaccination [5]. Nevertheless, chronic HBV infection remains a major global health concern. According to the World Health Organization (WHO), an estimated 254 million people were asymptomatic chronic HBsAg carriers in 2022, with 1.2 million new infections and 1.1 million HBV-related deaths each year [6].

Occult HBV infection (OBI) was first reported by Tabor et al. in 1979 [7]. They described a case of post-transfusion hepatitis B, acquired from blood from a donor whose only serological marker of HBV infection was anti-HBc [7]. For a long time thereafter, the existence of OBI remained controversial. However, with rapid advances in molecular technology and improved detection methods, an increasing number of studies have confirmed not only its existence but also its important clinical implications [8]. The transmission routes and clinical outcomes of OBI are the same as those of overt infection [9,10,11]. OBI can be found in individuals who are anti-HBc positive, and even in some who are only anti-HBs positive or who are completely negative for serological markers of HBV infection [12,13]. This poses a threat to the safety of donated blood and organs, and of immunosuppressive therapy.

The global prevalence of OBI in the general population is approximately 0.82%, although marked geographic and population-based disparities exist. Rates reach as high as 35.6% in high-endemic regions such as Uganda and 6.7% among healthcare workers in South Africa, compared to 0% in low-prevalence areas such as Switzerland [10,14,15]. The rate in the general population in China is 0.88% [15].

OBI is also common in populations vaccinated perinatally against HBV. The rate of OBI among vaccinated individuals in Taiwan is as high as 10.9% [16]. In a study in Jiangsu province, mainland China, the rate was 13.08% among anti-HBc-positive, vaccinated individuals, compared to 4.18% in the control group [17]. The rates among children born to HBsAg-positive mothers and immunised perinatally are 10.9% in Sweden and 5.6% in Shanghai, China, respectively [12,18].

We reported in 2025 that in LongAn County, China, the HBsAg positivity rate in the immunised population born between 1987 and 1993 was only slightly higher than the 2015 level (3.9% vs. 3.8%), whereas the rate of OBI had increased significantly (1.6% in 2015 vs. 6.0% in 2024) [19]. Here, based on the LongAn cohort, we used single-cell RNA sequencing (scRNA-seq) and a validation study to investigate the immunological characteristics associated with this phenomenon.

## 2. Materials and Methods

### 2.1. Study Subjects

The 15 subjects for scRNA-seq were selected from a cross-sectional study conducted in 2024 [19]. They were born between 1987 and 1993 and vaccinated perinatally with three 10 μg doses of plasma-derived vaccine. They were divided into three groups: Group 1 comprised five subjects with OBI, in whom OBI had not been detected in 2015. The factors for OBI diagnosis are described in our previous report [19]. Viral loads were measured in the same blood samples used for scRNA-seq, which were collected several months after those used for the initial OBI diagnosis. Group 2 comprised five ICHC subjects who were not infected with HBV in 2015 and were treatment-naïve. Their alanine aminotransferase (ALT) levels were below 40 U/L. Group 3 comprised five uninfected subjects, serving as normal controls. Each subject provided a 15 mL sample of blood for testing for serological markers of HBV infection and for scRNA-seq.

Informed consent in writing was obtained from each individual. The study protocol conforms to the ethical guidelines of the 1975 Helsinki Declaration and has been approved by the Guangxi Institutional Review Board.

### 2.2. Qualitative Assays of HBV Serological Markers

Sera were tested for HBV serological markers using enzyme immunoassays (WANTAI BioPharm, Beijing, China). Quality control for the measurements was performed in accordance with the protocols provided by the manufacturer.

### 2.3. Quantitative Assays of Anti-HBs

Serum anti-HBs concentrations were quantified by Maccura i6000, using HBsAb Quantitative Kits (Maccura Biotechnology Co., Ltd., Chengdu, China). According to the protocols provided by the manufacturer, positive and negative cutoffs were calculated using the positive and negative controls. The dynamic range of the kit is 4 mIU/mL~1000 mIU/mL anti-HBs.

### 2.4. Collection of Peripheral Blood Mononuclear Cells (PBMCs)

Two mL of the peripheral blood samples were collected into EDTA anticoagulation tubes and processed immediately. PBMCs were separated by density gradient centrifugation using Ficoll-Paque PREMIUM 1.084 g/L sterile solution (GE Healthcare, Uppsala, Sweden). Then, 90% foetal bovine serum +10% Dimethyl sulfoxide were added to the cryopreservation tube. The cells were stored at −80 °C prior to analysis.

### 2.5. Construction of Libraries and Single-Cell RNA Sequencing

Single-cell mRNA libraries were constructed using the DNBelab C Series Single Cell RNA Library Preparation Kit (MGI, Shenzhen, China), according to the protocol provided by the manufacturer. The water-in-oil droplets were prepared by adding a single-cell suspension, oil, and beads to a slide using the DNBelab C-TaiM instrument, completing mRNA capture and reverse transcription to generate cDNA. After demulsification, the cDNA was amplified, fragmented, end-repaired, A-tailed, adaptor-ligated, and PCR-amplified to construct the library; the oligo product was amplified, indexed, and purified. Both were denatured into single strands and circularised, then amplified by rolling circle amplification to form DNA nanoballs (DNBs), which were finally sequenced using combinatorial probe-anchor synthesis technology.

### 2.6. Quality Control and Determination of the Major Cell Types in the Single-Cell Data

The raw sequencing data were analysed to generate a gene expression matrix using DNBC4tools (version v2.1.1) [20]. Subsequent downstream analysis was conducted using the R package Seurat (version 5.3.0) [21]. Quality control of the cells was carried out according to the following criteria: (1) Cells with fewer than 200 genes identified or more than 90% of the maximum gene count were filtered out. (2) Cells were sorted in descending order based on the proportion of mitochondrial reads, and the top 15% were filtered out. Doublets were identified and removed using DoubletDetection [22]. Cell types were annotated by integrating markers from public databases (e.g., CellMarker, PanglaoDB, Cell taxonomy). Differential expression gene (DEGs) between groups were determined using the FindMarkers function (test.use = MAST).

### 2.7. Functional Analysis of Marker Genes or Differential Genes

GO and KEGG enrichment analyses were performed on differentially expressed genes using the org.Hs.eg.db (version 3.20.0) and clusterProfiler (version 4.14.6) packages in R (version 4.4.1). For KEGG analysis, the organism was set to “hsa” (human). Enriched terms/pathways with a *p*.adjust < 0.05 (Benjamini–Hochberg correction) were considered significant.

### 2.8. Gene Set Enrichment Analysis (GSEA) Analysis

GSEA was performed with the GSEA function of clusterProfiler (version 4.14.6) by ranking genes using the avg_log2FC from differential expression analysis. Gene sets were obtained from the MSigDB database (C2, C5, H) using the msigdbr package (version 25.1.0). The significance threshold was set at *p*.adjust < 0.05.

### 2.9. Functional Association Network and Protein-Protein Interaction (PPI) Network Analyses of HIPK2

PPI network analyses of HIPK2 GeneMANIA analysis was performed using the GeneMANIA database (version 3.6.0, https://genemania.org (accessed on 15 April 2026). HIPK2 was used as the query gene in Homo sapiens. All parameters were set to default values to generate a functional association network, predicting genes related to HIPK2 based on co-expression, physical interactions, genetic interactions, pathways, and shared protein domains.

STRING analysis was conducted using the STRING database (version 12.0, https://string-db.org) to construct a PPI network for HIPK2. The analysis was restricted to Homo sapiens, with a minimum interaction confidence score of ≥0.400 (medium confidence).

### 2.10. Measurement of Serum HIPK2 Protein

The concentration of serum homeodomain-interacting protein kinase 2 (HIPK2) protein was measured using an HIPK2 Enzyme-Linked Immunosorbent Assay Kit (Shanghai Meiao Biotechnology Co., Ltd., Shanghai, China). According to the protocols provided by the manufacturer, positive and negative cutoffs were calculated using the positive and negative controls. The dynamic range of the kit is 2~32 μmol/L and the sensitivity is 0.1 μmol/L.

### 2.11. Statistical Analyses

The Kolmogorov-Smirnov (K-S) test was used to assess overall differences in the distribution of pseudotime values between groups, focusing on shifts in the cumulative distribution. Continuous variables are expressed as means ± standard deviation (SD). Categorical data were evaluated using the χ^2^ test or Fisher’s exact test. Quantitative data were analysed using the independent samples *t*-test or the Mann–Whitney U test. Correlation analysis used the Pearson test or Spearman test, depending on the distribution of the data. All *p* values are two-tailed, and *p* < 0.05 is considered significant. R (version 4.4.1) and SPSS software (version 16.0; Chicago, IL, USA) were used for statistical analyses.

## 3. Results

### 3.1. General Characteristics of the Study Subjects

Fifteen subjects in total were recruited for scRNA-seq analysis, with five subjects in each group. All subjects in the OBI group were male, while there was one female in the ICHC group and two females in the control group. The mean ages in the OBI, ICHC, and control groups were 34.8 ± 2.8, 34.8 ± 2.2, and 34.8 ± 2.8 years, respectively (Table 1). Three subjects in the OBI group were positive for anti-HBs (>1000 mIU/mL). Viral loads in the OBI group were low, ranging from 132.43 IU/mL to 237.01 IU/mL.

### 3.2. Single-Cell Atlas of PBMCs from the Subjects with OBI

**Figure 1:**
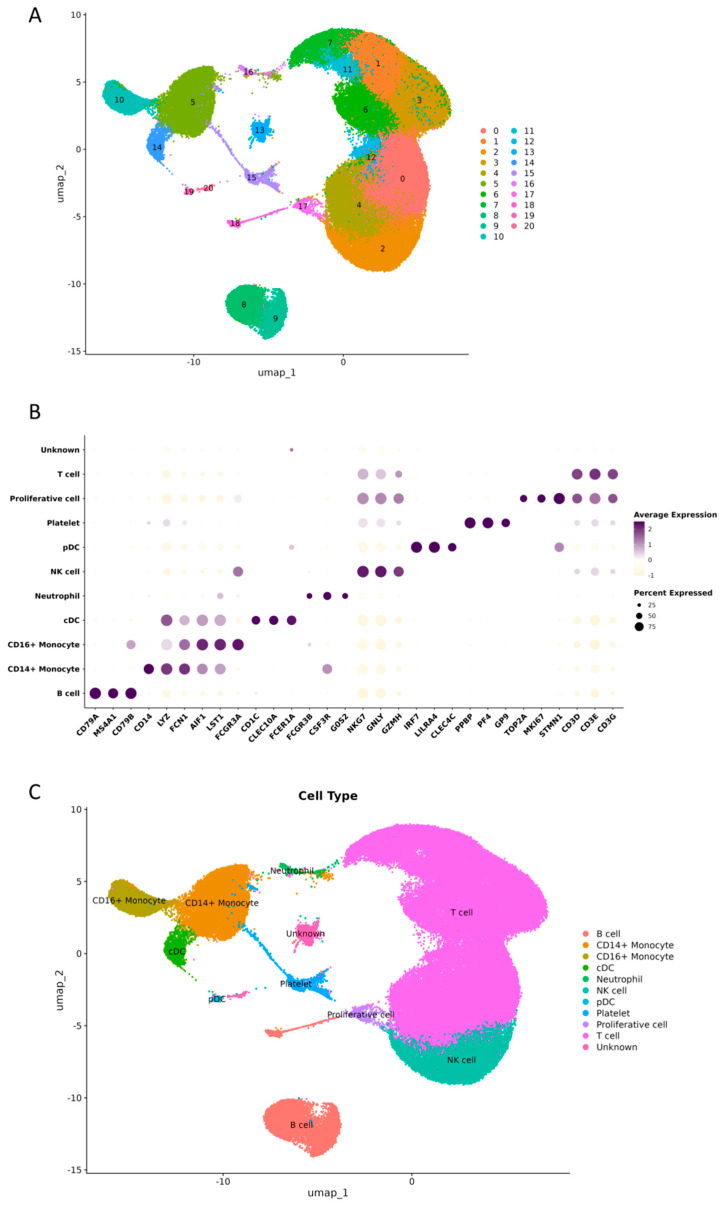
Single-cell transcriptome map of PBMCs from adults vaccinated against HBV at birth. (**A**) UMAP plot of the 122,958 single cells from 15 individuals, revealing 22 distinct cell clusters. All 15 samples were merged and analysed using Harmony for integration after normalisation, variable feature selection, and scaling with the Seurat package. The clusters were identified through unsupervised clustering and visualised using UMAP. (**B**) UMAP plot visualising the distribution of the 11 identified peripheral blood cell lineages. (**C**) Dot plots showing the expression of key marker genes (x-axis) in the major cell types (y-axis). The depth of the colour, from light to dark, and the size of the dots represent the average expression from low to high and the percentage of cells expressing the gene.

**Figure 2:**
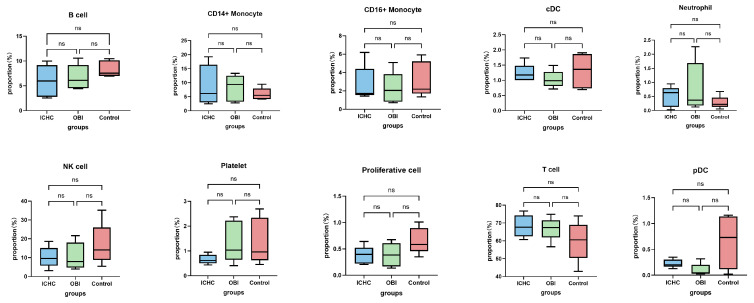
Box plot of cell type proportions in the three groups. The x-axis represents different cell types, and the y-axis represents the proportion. The boxes are coloured by groups. ns: not significant.

After quality control and filtering, 122,958 immune cells were obtained for single-cell RNA sequencing, including 42,431 cells from the OBI group, 40,808 cells from the ICHC group, and 39,719 cells from the control group. Twenty-one clusters were identified using unsupervised clustering (Figure 1A) and annotated as 11 cell types, based on canonical marker gene expression. Their distributions were visualised using Uniform Manifold Approximation and Projection (UMAP) (Figure 1B,C). The cell types included B cells (CD79A^+^MS4A1^+^CD79B^+^), CD14^+^ monocytes (CD14^+^LYZ^+^FCN1^+^), CD16^+^ monocytes (AIF1^+^LST1^+^FCGR3A^+^), conventional dendritic cells (cDCs) (CD1C^+^CLEC10A^+^ FCER1A^+^), neutrophils (FCGR3B^+^CSF3R^+^G0S2^+^), NK cells (FCGR3A^+^NKG7^+^GNLY^+^GZMH^+^), plasmacytoid dendritic cells (pDCs) (IRF7^+^LILRA4^+^CLEC4C^+^), platelets (PPBP^+^PF4^+^GP9^+^), proliferative cells (TOP2A^+^MKI67^+^STMN1^+^), T cells (CD3D^+^CD3E^+^CD3G^+^), and one unidentified cluster (Figure 1C). All cell types were present in the three groups, and no significant differences were observed among the groups in their proportions (Figure 2).

### 3.3. The Single-Cell Landscape of T Cells

**Figure 3:**
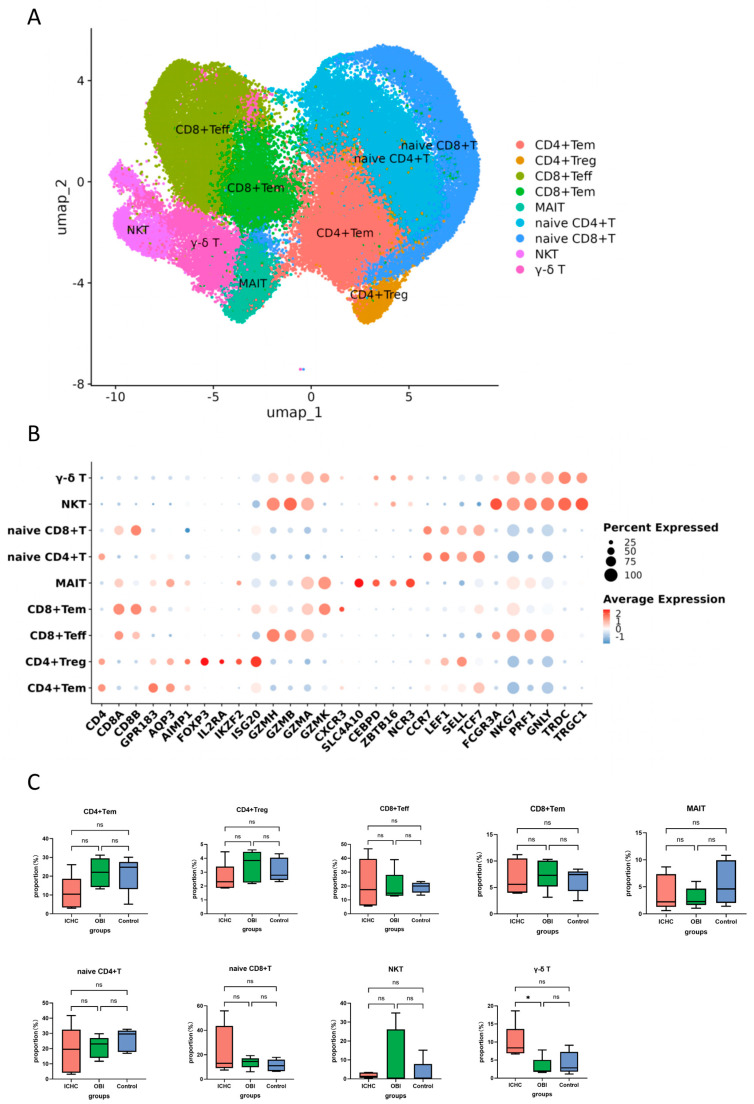
Single-cell transcriptome map of T cells. (**A**) UMAP plot visualising the distribution of the 9 identified subpopulations of T cells. (**B**) Dot plots showing the expression of key marker genes (x-axis) in the various cell types (y-axis). The depth of the colour, from light to dark, and the size of the dots represent the average expression from low to high and the percentage of cells expressing the gene. (**C**) Box plot of cell type proportions in the three groups. The x-axis represents various cell types, and the y-axis represents the proportion. The boxes are coloured by groups. *: *p* < 0.05. ns: not significant.

T-cell subsets were extracted from PBMCs for secondary clustering analysis. Nine T-cell subsets were identified based on marker gene expression (Figure 3A,B): naive CD4^+^ T cells (CD4^+^CCR7^+^LEF1^+^SELL^+^TCF7^+^), CD4^+^ T effector memory cells (CD4^+^GPR183^+^AQP3^+^AIMP1^+^), CD4^+^ regulatory T cells (CD4^+^FOXP3^+^IL2RA^+^IKZF2^+^ISG20^+^), naive CD8^+^ T cells (CD8A^+^CD8B^+^CCR7^+^LEF1^+^SELL^+^TCF7^+^), CD8^+^ T effector cells (CD8A^+^CD8B^+^GZMH^+^GZMB^+^ GZMA^+^), CD8^+^ T effector memory cells (CD8A^+^CD8B^+^GPR183^+^GZMA^+^GZMK^+^CXCR3^+^), MAIT cells (SLC4A10^+^CEBPD^+^ZBTB16^+^NCR3^+^), NKT cells (CD3D^+^CD3E^+^CD3G^+^FCGR3A^+^ NKG7^+^PRF1^+^GNLY^+^), and γδ T cells (TRDC^+^TRGC1^+^). The composition and number of T-cell subsets were consistent across the OBI, ICHC, and control groups. No significant differences were found in the proportions of T-cell subsets among the three groups (Figure 3C).

### 3.4. Downregulated Expression of the HIPK2 Gene Is Associated with OBI

Differentially expressed gene (DEG) analysis was performed on T cells and B cells from the three groups. We identified 1481, 6403, and 7057 DEGs in T cells for the OBI vs. control, OBI vs. ICHC, and ICHC vs. control comparisons, respectively. For the B cells, the corresponding numbers were 495, 659, and 1221.

**Figure 4:**
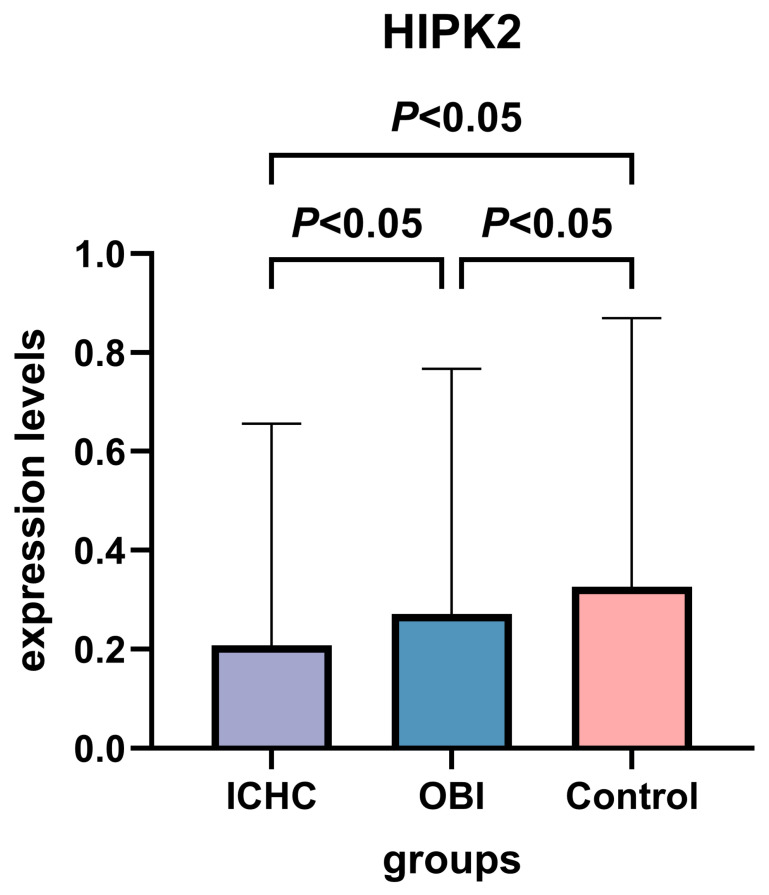
Differential gene expression (DGE). Differential expression of the HIPK2 gene in T cells among the three groups (Mann–Whitney U test).

HIPK2 exhibited a unique differential expression pattern in T cells that was strongly associated with the OBI state. In the OBI group, HIPK2 expression was downregulated 1.42-fold compared to the control group (*p* = 2.01 × 10^−69^). In contrast, it was upregulated 1.34-fold compared to the ICHC group (*p* = 9.95 × 10^−61^) (Figure 4). These results indicate that HIPK2 expression in T cells is strongly suppressed in ICHC and less so in OBI, compared to healthy controls. This distinctive pattern suggests HIPK2 expression may serve as a specific marker of the immune state in occult HBV infection.

### 3.5. DEGs Enrichment Analysis

**Figure 5:**
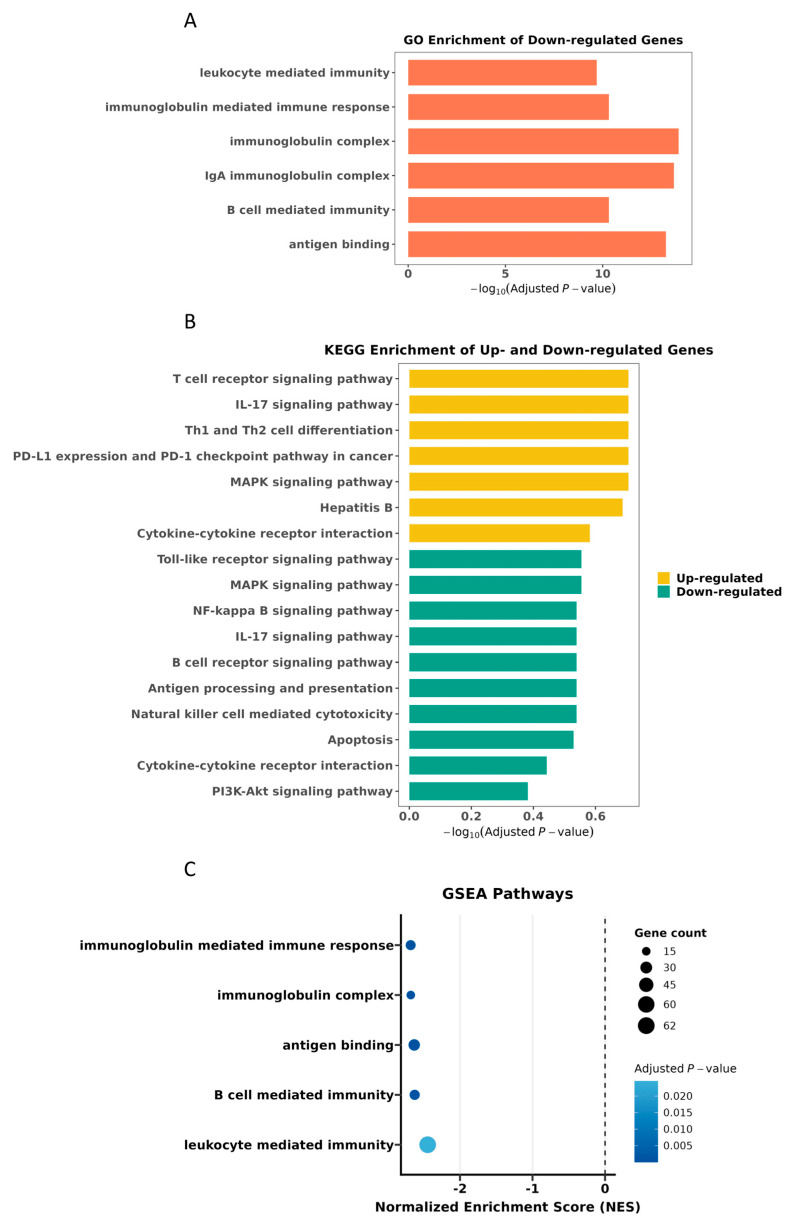
Differential gene expression (DGE) functional enrichment. (**A**) GO enrichment analysis of differentially expressed genes in T cells between the OBI and healthy control groups. (**B**) KEGG enrichment analysis of differentially expressed T-cell genes between the OBI and healthy control groups. (**C**) Gene set enrichment analysis (GSEA) pathways enriched by differentially expressed genes. The x-axis represents the normalised enrichment score (NES), and the y-axis shows the names of the enriched pathways. The size of each circle indicates the number of genes (Gene count) contained in the pathway, while the colour of the circle represents the adjusted *p*-value. GSEA of differentially expressed genes in occult HBV infection (OBI) compared with normal controls revealed focused enrichment in humoral immunity and B cell-mediated immune responses. Unlike standard over-representation analysis, GSEA identified coordinated changes across entire gene sets, indicating that these immune processes are systematically altered in OBI.

Functional enrichment analysis of DEGs was performed using the GO and KEGG pathway databases. In the GO analysis (covering biological processes, cellular components, and molecular functions), 2 DEGs were upregulated and 34 were downregulated. In the KEGG analysis, 87 DEGs were upregulated and 59 were downregulated. GO and KEGG enrichment results from DEGs in the OBI group highlighted strong enrichment in immune response-related processes and signalling pathways (Figure 5A,B). These findings suggest significant immune system activation or dysregulation in response to the persistent, low-level viral presence in OBI. The enhanced expression involved both innate and adaptive immunity, antigen recognition, immune cell signalling, cytokine networks, and cell fate decisions (e.g., apoptosis and immune checkpoints).

### 3.6. Protein–Protein Interaction Scores Between Hipk2 and Selected Partners

**Figure 6:**
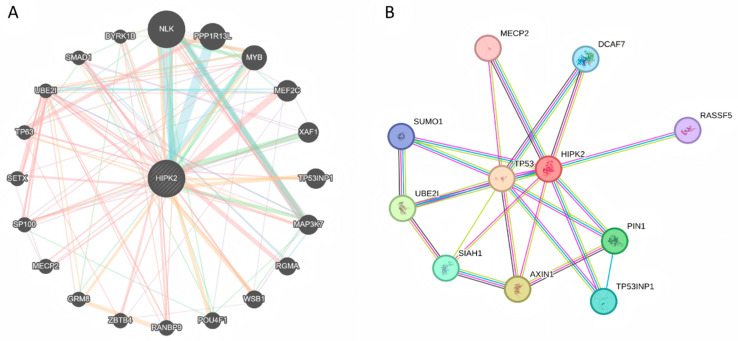
Functional association network and protein-protein interaction (PPI) network analyses of HIPK2. (**A**) The GeneMANIA high-confidence functional network of HIPK2. Nodes represent genes/proteins (the striped node = HIPK2). Edge colours represent various types of functional associations. (**B**) The STRING protein–protein interaction network of HIPK2. Nodes represent proteins (the striped node = HIPK2). Line colours indicate different types of evidence for interactions.

Network analysis of HIPK2 using the STRING and GeneMANIA databases revealed extensive protein–protein interactions. In the high-confidence GeneMANIA network (Figure 6A), HIPK2 formed a compact module with TP53 at the centre, showing strong direct interactions with key regulators, including TP53INP1, PIN1, AXIN1, SIAH1, UBE2I, SUMO1, RASSF5, DCAF7, and MECP2. In contrast, the broader STRING interaction network (Figure 6B) positioned HIPK2 as a highly connected hub, with numerous functional associations with proteins involved in p53 signalling (e.g., TP53INP1, PPP1R13L, TP63), transcriptional regulation (e.g., MYB, MEF2C, POU4F1, ZBTB4, MECP2), stress-response kinases (e.g., NLK, MAP3K7, DYRK1B), and other pathways such as SUMOylation, ubiquitination, TGF-β/SMAD, and Wnt/β-catenin signalling. These findings indicate that HIPK2 serves as a central signalling node, integrating multiple cellular stress and regulatory pathways, with a prominent role in the TP53 network.

### 3.7. Low Concentrations of Serum Hipk2 Protein Were Associated with OBI

Serum HIPK2 protein levels were measured in the samples used for single-cell RNA sequencing. No significant difference was observed in the mean serum HIPK2 concentrations between males (128.6 ± 144.9 μmol/L) and females (132.9 ± 172.5 μmol/L) (*p* = 0.734), indicating that sex may not influence HIPK2 protein levels. The mean serum HIPK2 concentration in the OBI group (27.1 ± 4.9 μmol/L) was significantly lower than that in the normal control group (35.4 ± 5.4 μmol/L) (*p* = 0.047), consistent with the single-cell RNA sequencing results. However, the OBI group showed a significantly lower serum HIPK2 concentration than the ICHC group (326.0 ± 13.6 μmol/L) (Table 1). This finding contrasts with the gene expression pattern observed in single-cell RNA sequencing.

## 4. Discussion

To the best of our knowledge, this is the first study to demonstrate an association between HIPK2 and OBI. The major finding is that downregulated HIPK2 gene expression in T cells is associated with OBI. HIPK2 expression levels in both T cells and serum were significantly lower in the OBI group than in the healthy control group. DEGs identified in OBI were broadly involved in immune response pathways, with GO and KEGG analyses showing strong enrichment in innate and adaptive immunity, antigen presentation, cytokine signalling, and cell fate decisions. GSEA further revealed focused and systematic enrichment of humoral immunity- and B cell-mediated gene sets. Within the molecular network associated with OBI, HIPK2 functions as a central signalling hub, with TP53 playing a critical role in mediating its interactions. The main strength of this study is the concordance between the single-cell transcriptomic downregulation of HIPK2 in T cells and the reduced HIPK2 levels observed in peripheral serum. However, this study has several limitations. First, these findings have not been confirmed in independent cohorts. Second, serum HIPK2 protein levels were measured in total serum rather than in isolated T cells. This limitation prevents us from determining whether the reduced serum HIPK2 concentration reflects decreased T cell-derived protein expression or post-translational regulation. Another limitation is the small number of female participants, which precludes a definitive conclusion regarding sex differences in serum HIPK2 levels. Furthermore, the lack of contemporaneous viral load data from the ICHC group limits the interpretation of the relationship between immune signatures and viral replication status.

Few studies have explored the pathogenesis of OBI using immune cell transcriptome profiling (e.g., RNA-seq or microarray analysis of PBMCs, T cells, or other lymphocytes). OBI research has more commonly focused on viral factors (such as mutations, integration, and epigenetics), host immune control (particularly T-cell responses), and broader multi-omics approaches, rather than single immune cell transcriptomics [10,23]. One recent study highlighted the association of CD4^+^ T effector memory cells and memory B cells with OBI, suggesting their roles in rapid responses to reactivation through cytokine production and antibody secretion [24]. That study also incorporated tissue tracing and machine learning models of differentiation. Zerbini et al. examined HBV-specific T-cell responses in OBI patients and distinguished distinct profiles based on the anti-HBc status, with protective memory-like responses in seropositive patients and weaker responses in seronegative patients, supporting the hypothesis of low-dose infection [25]. Although that study focused on functional immunology rather than full transcriptome analysis, it provided valuable insights into immune control in OBI. The present study is the first to characterise the full transcriptome of PBMCs at single-cell resolution in OBI. This approach offers a comprehensive view of the unique “immune-virus balance” in OBI and may provide new insights into viral latency mechanisms, as well as potential therapeutic targets and biomarkers.

The HIPK2 gene encodes a serine/threonine kinase that plays a central role in regulating gene transcription, cell growth, and apoptosis, particularly in response to DNA damage [26]. HIPK2 also regulates STAT3 transcriptional activity in a cell-type-specific manner during Th17 cell differentiation [27]. In the context of viral infection, HIPK2 is essential for the production of type I interferons. HIPK2-deficient macrophages produce significantly lower levels of type I interferons following vesicular stomatitis virus (VSV) infection, and HIPK2-deficient mice are more susceptible to lethal VSV disease than wild-type mice [28].

HIPK2 is known to stabilise and activate TP53 by reducing Mdm2 protein levels, thereby enhancing TP53 transcriptional activity [29]. It has also been reported that TP53 downregulates the HBV regulatory protein HBx through ubiquitin-dependent proteasomal degradation involving E6AP, which suppresses viral transcription and replication in HCC cells [30]. Disruptions in TP53 signalling pathways have been linked to HBV persistence, reactivation risk, and progression to cirrhosis or HCC, even in patients with OBI [31,32]. We therefore hypothesise that downregulated HIPK2 expression reduces TP53 transcriptional activity, leading to sustained low-level HBV transcription and replication. This mechanism may help maintain the occult state of infection, preventing both viral clearance and progression to overt chronic hepatitis B. In vaccinated individuals with pre-existing downregulated HIPK2 expression, repeated long-term exposure to HBV, due to ageing and increased opportunities for exposure, can lead to OBI. Consequently, the prevalence of OBI increases with age among those vaccinated at birth. However, our hypothesis is based on transcriptomic association and network analysis, rather than a demonstrated causal mechanism. Functional experiments are needed to support this claim.

OBI shares the same transmission routes as overt HBV infection. However, due to its occult nature (HBsAg-negative), it can occur in individuals who test negative for all HBV serological markers or who are positive only for anti-HBs. Consequently, the presence and potential harm of OBI are often overlooked, posing significant challenges for prevention and control [33]. In this context, our findings may not only offer transcriptome-derived insights into the unique “immune–virus balance state” in OBI but also provide potential biomarkers of OBI.

mRNA–protein discordance is common, particularly for kinases such as HIPK2. HIPK2 is heavily regulated at both the post-transcriptional and post-translational levels. Its protein stability and activity are tightly controlled, especially under conditions of cellular stress or viral infection [34]. In T cells, factors such as translation efficiency, microRNA regulation, and signalling triggered by HBV antigens or cytokines can decouple mRNA levels from protein output. Indeed, studies of immune cells have frequently reported poor mRNA–protein correlations due to rapid translational control during cell activation and differentiation [35].

Furthermore, scRNA-seq captures intracellular mRNA in specific T-cell populations at a single time point, reflecting local transcriptional regulation in circulating or tissue-resident T cells. In contrast, serum ELISA measures the total circulating HIPK2 protein, which can originate from multiple cell types and tissues (not only T cells), from cell death, secretion, or leakage. In overt HBV infection, higher levels of inflammation, hepatocyte damage, and cellular stress may promote greater protein release into the bloodstream, resulting in elevated serum HIPK2 levels [32,34]. These differences help explain our findings: serum HIPK2 levels were significantly lower in the OBI group than in the normal control group, consistent with the scRNA-seq results, whereas the OBI group also showed significantly lower levels than the ICHC group, which contrasts with the scRNA-seq data.

It remains unclear whether the lower serum HIPK2 protein levels reflect reduced production by T cells or result from post-translational regulation, and whether they are linked to the Th17/STAT3 pathway. Although HIPK2 is known to play a critical role in Th17 lineage-specific regulation of STAT3, it is uncertain whether the differences in HIPK2 expression seen between OBI and ICHC support controlled antiviral responses without triggering excessive inflammation.

To address these questions, we plan to perform intracellular HIPK2 protein quantification in T-cell subsets using flow cytometry or CyTOF, comparing samples from OBI patients, ICHC patients, and healthy controls. We will also conduct phospho-specific staining to assess activated HIPK2 and downstream *p*-STAT3 (Ser727) in the same cells. In addition, to explore the clinical and translational value of HIPK2, we will perform longitudinal monitoring of serum HIPK2 levels in OBI patients to determine whether this can predict viral reactivation or resolution.

## 5. Conclusions

We demonstrated that downregulated HIPK2 expression occurred in T cells, and lower HIPK2 protein levels in serum, in OBI patients than in healthy controls. Transcriptomic analyses revealed broad immune response activation, with strong enrichment in humoral and B cell-mediated immunity. Network analysis identified HIPK2 as a central signalling hub largely mediated by TP53, suggesting that reduced HIPK2 may impair TP53 activity and promote low-level HBV persistence. These findings suggest that HIPK2 levels are a feature of OBI and may provide new insights into its pathogenesis. Future validation in independent cohorts and functional studies is warranted to confirm its clinical utility.

## Figures and Tables

**Table 1:** Baseline information of the study subjects.

Code	Group	Gender	Age	HBsAg	Anti-HBs (mIU/mL)	HBeAg	Anti-HBe	Anti-HBc	HBV DNA (IU/mL)	HIPK2 (μmol/L)
A1	OBI	M	37	-	0	-	-	-	227.431	33.439
A7	OBI	M	36	-	0	-	-	-	144.763	30.87
B4	OBI	M	34	-	>1000.000	-	-	-	216.221	24.825
B7	OBI	M	36	-	>1000.000	-	-	+	237.01	21.395
B9	OBI	M	31	-	>1000.000	-	-	-	132.427	24.995
**Average**										**27.1048**
D1	ICHC #	M	37	+	0	-	+	+	ND *	333.785
D10	ICHC	M	37	+	0	-	+	+	ND	332.335
D2	ICHC	M	32	+	0	-	+	+	ND	329.79
D3	ICHC	F	34	+	0	-	+	+	ND	332.155
D8	ICHC	M	34	+	0	-	-	+	ND	301.78
**Average**										**325.969**
A2	Control	F	36	-	0	-	-	-	0	32.425
A3	Control	M	31	-	0	-	-	-	0	30.07
A4	Control	F	36	-	0	-	-	-	0	34.205
A5	Control	M	35	-	0	-	-	-	0	44.305
A6	Control	M	36	-	0	-	-	+	0	35.825
**Average**										**35.366**

* ND: not done. #: ICHC: inactive chronic HBsAg carriers.

## Data Availability

The datasets used and analysed during the current study are available from the corresponding author upon reasonable request.

## Abbreviations

anti-HBc	hepatitis B core antibody
anti-HBs	hepatitis B surface antibody
avg_log2FC	average log2 fold change
cDC	conventional dendritic cells
cDNA	complementary DNA
DEGs	differentially expressed genes
DNBs	DNA nanoballs
GO	gene ontology
GSEA	gene set enrichment analysis
HBV	hepatitis B virus
HBsAg	hepatitis B surface antigen
HCC	hepatocellular carcinoma
HIPK2	homeodomain-interacting protein kinase 2
ICHC	inactive chronic HBsAg carriers
KEGG	Kyoto Encyclopedia of Genes and Genomes
K-S test	Kolmogorov-Smirnov test
MAIT	mucosal-associated invariant T cells
mRNA	messenger RNA
NES	normalized enrichment score
NK cells	natural killer cells
OBI	occult HBV infection
PBMCs	peripheral blood mononuclear cells
PCR	polymerase chain reaction
pDC	plasmacytoid dendritic cells
PPI	protein–protein interaction
scRNA-seq	single-cell RNA sequencing
SD	standard deviation
STAT3	signal transducer and activator of transcription 3
TP53	tumour protein p53
UMAP	uniform manifold approximation and projection
VSV	vesicular stomatitis virus
